# Evaluation of radiological hazards associated with some Egyptian marble and granite rocks

**DOI:** 10.1038/s41598-024-80298-1

**Published:** 2024-11-21

**Authors:** M. A El-Nahal, Mahmoud K. Alawy, Mohamed Elsafi

**Affiliations:** 1 Department of Environmental Studies, Institute of Graduate Studies and Research, Alexandria University, Alexandria, 21511 Egypt; 2 Geology Department, Faculty of Science, Alexandria University, Alexandria, 21511 Egypt; 3 Physics Department, Faculty of Science, Alexandria University, Alexandria, 21511 Egypt

**Keywords:** Natural radioactivity, Building materials, Radiation hazard parameters, Marble, Granite, NORM, Geochemistry, Potassium feldspar., Environmental sciences, Solid Earth sciences, Physics

## Abstract

The aim of the current study is to evaluate the radioactivity estimate the radiological risk of some granites and marbles rocks and explaining the cause of increased radioactivity in some types of rocks. The radioactivity of some granites and marbles produced in Egypt were determined by using a Germanium detector. Three types of marble (Breshia, Galala, and Trista) and three types of granite (Gandola, White Halayeb, and Red Aswani) were collected. All marble samples show low radioactivity with average activity concentrations of 20 ± 2, 4.50 ± 0.5, and 6.70 ± 1.2 Bqkg^− 1^ for ^226^Ra, ^232^Th, and ^40^K respectively. Granite samples have higher activity concentration with averages of 152 ± 7, 129 ± 8, and 1228 ± 15 Bqkg^− 1^ for ^226^Ra, ^232^Th, and ^40^K respectively which exceed the world average values of soil (32,45,412 Bqkg^− 1^ for ^226^Ra, ^232^Th, and ^40^K respectively) excluding Granite G.2 (white Halayeb) as it shows an insignificant level of radioactivity. The annual effective doses of marble samples Breshia, Galala, and Trista were measured to be 4.42 ± 0.4; 158 ± 14 and 153 ± 15 µSvy^− 1^, and 1008 ± 147, 80 ± 7 and 987 ± 45.0 µSvy^− 1^ for the granite samples Gandola, White Halayeb and Red Aswani respectively. The radiation hazard parameters show a higher value for granite samples than marble samples, primarily due to the presence of potassium feldspar minerals in these types of granites. marbles were observed to be radiologically safer than granite because they possess a neglected ^40^K content and a trace quantity of uranium and thorium. Moreover, the minimum potassium content is enough to make a rock radiological unsafe due to ^40^K only being determined to be about 13.2%.

## Introduction

Marbles and Granites are widely used as building materials and decorative tiles in most modern residents and workplaces, hence the radiological hazard associated with those tiles could have a potential adverse effect on human health^1^.

Dolomite or limestone is the starting material for marble, which has a granular rock texture. It consists of several interconnected calcite or dolomite grains. Marbles are formed when the pressure and temperature of a massive drop combine over limestone buried deep within the Earth and may result from contact metamorphism near igneous intrusions. During metamorphism limestone impurities may recrystallize into marble into mineral impurities, mostly iron oxides, graphite, and mica^2^.

Granite is the most common rock intrusion on Earth’s continental shelf. It is commonly referred to as an ornamental stone with pink, white, white, and black blocks. It has medium to coarse grains. The three main minerals are quartz, mica (which can be found as black biotite or silvery scovite), and feldspar. These contain quartz, often over 10%. Because alkali feldspars tend to be pink in color, pink granite is often used as a decorative stone. Silica-rich magmas, many kilometers underground produce granite crystals^3^.

The NORM which is the naturally occurring radioactive materials (^238^U decay series, ^235^U decay series, ^232^Th decay series, and ^40^K) may find a way to accumulate or disperse through this rock during the stages of rock formation^4,5^. Some are classified as mineral-bearing uranium like quartz and feldspar. The traces of uranium and thorium decay series contained in the minerals forming rocks contributed to the radiation exposure from these rocks, in addition to the radiation emitted by potassium-40 (^40^K) which is a naturally occurring radioactive isotope of elemental potassium. The natural abundance of K-40 is 0.0117%^6^.

Many previous research examines a variety of marbles and granites across the globe, they estimate their radiological hazard indices and radium equivalent values. The common missing part in all those studies is that there was no sufficient explanation as to why some rocks are more radioactive than others. There is no analytical comparison between marble and granite according to the radiological safety perspective. Another deficiency associated with most of that research is that they depend on the commercial classifications of the rocks which is sometimes not accurate so this deficiency will be rectified by performing a material characterization analysis to confirm whether these rocks are being marbles or granites or not^7–12^. Several studies have been conducted on the radioactivity of marble, granite and some rocks in Egypt. These studies aim to evaluate the levels of natural radiation in these materials and their safe use in construction^13–18^.

The importance of the current study arises from the chronic effects of ionizing radiation on human health. Using radiologically unsafe stones as a building block may increase the probability of developing cancer in human inhabitants hence commercially building stone and decorative tiles must be carefully filtered according to their radiological properties. In this research, the most widely used types of marble and granite in Egypt market will be chemically investigated and radiologically evaluated.

the current study presents a comprehensive cause analysis radiologically comparison between some selected types of Egyptian marbles and granites where not only the radiological hazard parameters will be estimated and compared but also will answer questions such as why some types of rocks are less radiological safer than others so it can be established some radiological selection criteria to filtrate these types of rocks before using it as building materials or decorative tiles.

## Materials and methods

The most common marble and granite types in the Egyptian market were geochemically investigated to confirm their commercial calcification prior to the radiological examination of the samples.

### Sample collection

The samples were collected from the Egyptian market; three types of marble and three types of granite were collected. All these types were produced from different quarry sites in Egypt. Ten samples of each type were collected. All samples of the same type were grouped and marked by a sample group serial number. Serial number, the Type, the commercial name, and the region of the production site in Egypt of the samples are described in Table 1.

**Fig. 1:**
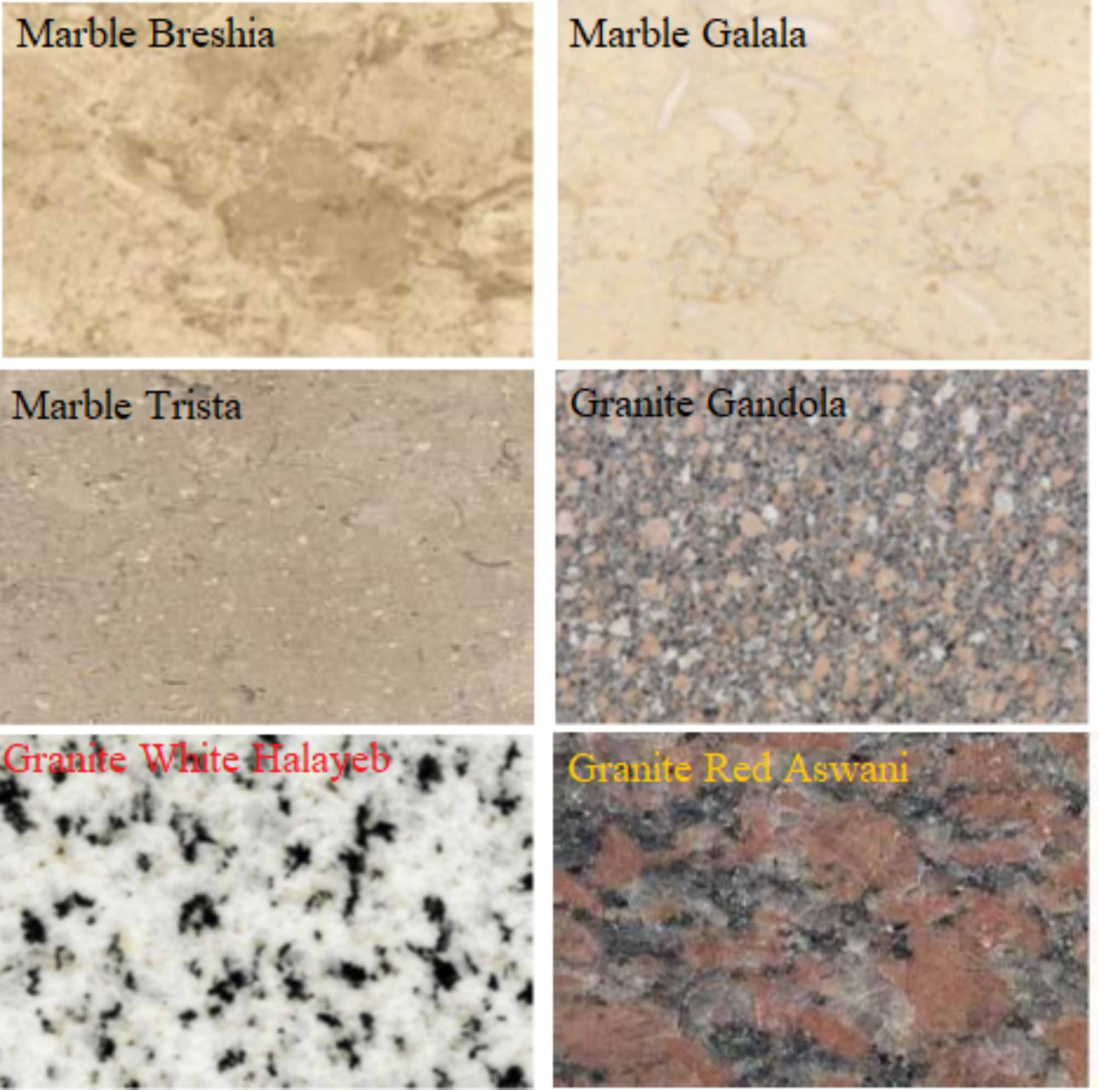
Different types of granite and marble types.

The surface shape and texture of the samples under consideration after processing and polishing are shown in the Fig. 1.The types of granites and marbles under investigation in our study are very common in the Egyptian market as decoration or building stones, the popularity of the stones represented the selecting criteria of materials that will be studied in this research.

### Sample preparation for Energy Dispersive X-ray (EDX) measurement

The samples were prepared and processed to execute the radiation measurements and analytical measurements by the Energy Dispersive X-ray (EDX) unit of the electron microscope to identify the samples’ chemical compositions and confirm that commercial classifications of the rocks being marbles or granites are true. To carry out the material characterization testing, samples in powder form were coated with a thin layer of gold under a vacuum before be investigated by the EDX unit of the scanning electron microscope (SEM) (Model: JSM-5300, JEOL) to determine the chemical compositions of the samples under study.

### Sample preparation for radioactivity measurement

To carry out the radioactivity measurement, the samples should be reduced to be in the form of homogeneous powder of the material so that each sample was grinded and processed into a powder then sieved a 200 mesh and dried in an oven at a temperature between 105 and 110 °C to eliminates the moisture, the final powder sample weight was 200 g only for each one to reduce the sample self-absorption then it was firmly enclosed inside 250 mL Marinelli beaker to achieve approximately 4Π good detection geometry, they will be kept for 4 weeks till the secular equilibrium between all members of each decay series will be established.

### Sample analysis

The radiation measurements were carried out by using Canberra (Mirion) high purity germanium (HPGe) gamma-ray detector (model CS20-A31CL) equipped with a lead shield at the central lab of radiation measurements of Alexandria University, Egypt. The detector energy calibration was performed by using ^241^Am (59.54 keV), ^137^Cs (661.9 keV), and ^60^Co (1173.2 and 1332.5 keV) radioisotopes point sources and the detector efficiency curve was established by using ^152^Eu source in the powder form to resemble the sample matrix. The accumulation times of radiation standards measurements were sufficient to acquire at least 10^4^ counts under each photopeak of the standard sources to reduce the statistical error. The spectra were processed by Genie 2000 software (V3.1) released by Canberra (Mirion) Company (Genie™ Spectroscopy Software Suite - Mirion). Finally, the minimum detectable activities of the interested radionuclides exist in the samples listed in Table 2; these radioisotopes significantly contribute to the radiation doses from the material under study. The minimum detectable activity (MDA) is related to detector sensitivity and can be defined as the smallest amount of activity distinguishable from a background which can be quantified at a given confidence level (usually 95%), the minimum detectable activity is automatically calculated by Canberra (Mirion) Genie 2000 (V3.1) software (Genie™ Spectroscopy Software Suite - Mirion). The energy resolution (FWHM) obtained from the measurements is about 1.8 keV at the 1.33 MeV of the ^60^Co gamma lines.

**Table 1:** The sample’s marking and descriptions.

Sample Code	Type of the samples	Commercial name	Production site region
M.1	Marble	Breshia	Sinai
M.2	Marble	Galala	Suez
M.3	Marble	Trista	Sinai
G.1	Granite	Gandola	Sinai
G.2	Granite	White Halayeb	Halayeb
G.3	Granite	Red Aswani	Aswan

**Table 2:** The minimum detectable activity radionuclides of interest.

Decay series	^238^U Series				^232^Th Series						^40^K
Radioactive isotope	^234^Th	^226^Ra	^214^Bi	^214^Pb	^228^Ra (^228^Ac)		^234m^Pa	^212^Pb	^212^Bi	^208^Tl	^40^K
Energy (keV)	92	186	609	351	911	968	1001	238	727	583	1460
Minimum detectable activity (Bqkg^− 1^)	0.27	9.51	1.45	3.38	5.1	8.75	1.87	2.19	1.45	0.92	3.571

The sample measurement time should be selected to be as long as possible because some samples are expected to record very low levels of radioactivity. It was found that 48 h. is the optimum time for measurements of all samples. The secular equilibrium for all decay series was assumed to apply to all samples, ^226^Ra activity concentration (C_Ra_) was estimated from the average concentrations of the ^214^Pb (351.9 keV) and ^214^Bi (609 and 351 keV) decay products, and eventually ^232^Th specific activity (C_Th_) can be calculated by 911 keV gamma line of its daughter ^228^Ac. A ^40^K specific activity can be measured directly through a ^40^K gamma line of 1460 keV.

Since ^40^K is a common background radioisotope hence an accurate determination of background before any radiation measurement is essential, so besides using the low background measurement system the counting time of background was long enough to achieve obvious spectra of the interested background gamma lines. It was found that the optimum background counting time by the high-purity germanium (HPGe) detector in this research is about 7 days considering that the relative efficiency of the detector is about 20%. Empty Marinelli breakers were used to perform the background measurements.

The specific activities of the samples and their associated errors were calculated according to the following equations^19^:

1 $${{\text{A}}_{\text{s}}}({\text{Bqk}}{{\text{g}}^{ - 1}})=\frac{{{R_s}}}{{{\mathbf{E}}ff\left( {\mathbf{E}} \right) \times {\mathbf{P\gamma }}\left( {\mathbf{E}} \right) \times {\mathbf{M}}}}$$

2 $${\text{Uncertainty}}, ({\text{BqK}}{{\text{g}}^{{\text{-1}}}})= \pm \sqrt {\frac{{{\text{Rs}}+{\text{Rg}}}}{{{\text{Ts}}}}+\frac{{{\text{Rg}}}}{{{\text{T}}g}}}$$

A_s_ represent the specific activity of each nuclide in (Bqkg^− 1^), R_s_ is the count per second after background subtracting, ${\mathbf{Eff}}(\mathbf{E})$ the efficiency of the detector of gamma-ray, P_γ_ is the gamma decay probability (gamma transition Probability), M represent the mass of the sample, R_g_ is the background count rate, T_g_ equal background counting time and T_s_ is the sample counting time.

### Radiological hazard indices

The radiological hazard indices are Radiation Hazard standard parameters utilized to evaluate the consequences of radiation exposure on the health of people and the environment. These indices are useful in estimating the potential radiological influence of samples that contain radionuclides (^238^U, ^232^Th, and ^40^K) by a single parameter, which takes into consideration the radiation hazard associated with them. The indices and their corresponding equations are presented below.

The annual effective dose equivalent (AEDE) received indoors by an individual from a building material containing natural radioactivity is calculated from the absorbed dose rate by introducing the dose conversion factor of 0.7 SvGy^− 1^. Considering that individuals on average spend 80% of their lifetime indoors, the occupancy factor of indoors is 0.8^20^. AEDE is calculated by the equations below. The AEDE (indoor) occurs within a residence where the radiation risks because of building materials only are taken into consideration^21^.

3 $${\text{AEDE }}\left( {{\text{Indoor}}} \right) ({\text{mSv}}{{\text{y}}^{ - 1}})\,=\,{\text{Absorbed dose D }}({\text{nGy}}{{\text{h}}^{ - 1}}) \times {\text{876}}0{\text{ h}} \times 0.{\text{7 SvG}}{{\text{y}}^{{\text{-1}}}} \times 0.{\text{8}} \times {\text{1}}{0^{{\text{-3}}}}$$

In order to determine the annual effective dose equivalent, we have to estimate the absorbed dose in the air first according to Eq. (1). the air absorbed dose rate (nGyh^− 1^) from a building material due to the average specific activity concentrations of ^238^U, ^232^Th, and ^40^K in (Bqkg^− 1^) was determined by using the following Eqs^22,23^:

4 $${\text{D}}=0.{\text{427}}{{\text{C}}_{\text{U}}}+0.{\text{662}}{{\text{C}}_{{\text{Th}}}}\,+\,0.0{\text{432}}{{\text{C}}_{\text{K}}}$$

C_U_ is the average specific activity of ^238^U, C_Th_ is the average specific activity of ^232^Th and C_k_ is the average specific activity of ^40^K in samples. This expression determines the absorbed dose rate in the air at 1.0 m from the land due to the measured radionuclide concentration in the environmental materials.

The upper limit of radiological dose due to building materials is 1.0 mSv/y^24^. To restrict the dose to that limit, the following conservative model established on a wall of infinite thickness and free of windows and doors to use as a criterion for the estimation of external hazard index H_ex_ is given by^23^:

5 $${{\text{H}}_{{\text{ex}}}}=\frac{{{C_{Ra}}}}{{370}}+\frac{{{C_{Th}}}}{{259}}+\frac{{{C_k}}}{{4810}}$$

The value of this index must be less than unity for the radiation hazard to be negligible so that the radiation exposure due to radioactivity in construction materials must be limited to 1.5 mSv/y. In addition to external irradiation, radon, and its short-lived products are also hazardous to the respiratory organs. The internal exposure to radon and its daughter products is quantified by the internal hazard index (H_in_) which is given by the following equation^25^.

6 $${{\text{H}}_{{\text{in}}}}=~\frac{{{C_{Ra}}}}{{185}}+\frac{{{C_{Th}}}}{{259}}~+\frac{{{C_k}}}{{4810}}$$

C_Ra_, C_Th,_ and C_k_ are the average specific activity of ^226^Ra, ^232^Th, and ^40^K respectively in Bqkg^− 1^ for the material. For the safe utilization of the material in the construction of houses H_in_ should be less than unity. Finally, in order to compare the specific activity of materials containing different amounts of ^226^Ra, ^232^Th and ^40^K, we should use the radium equivalent activity (Ra_eq_) in Bqkg^− 1^ which can be determined through the following relation^26^:

7 $${\text{R}}{{\text{a}}_{{\text{eq}}}}={{\text{C}}_{{\text{Ra}}}}+{\text{1}}.{\text{43}}{{\text{C}}_{{\text{Th}}}}\,+\,0.0{\text{77}}{{\text{C}}_{\text{K}}}$$

It is assumed that 370 Bqkg^− 1^ of ^226^Ra, 259 Bqkg^− 1^ of ^232^Th, and 4810 Bqkg^− 1^ of ^40^K generate the same gamma-ray dose rate^25,26^. A radium equivalent of 370 Bq/kg in construction materials will generate an exposure of about 1.5mSv/y to the resident^28^. The recommended maximum levels of radium equivalents for construction materials to be utilized for dwelling are 370 Bq/kg and for industries are 370 to 740 Bqkg^− 1^^29^.

## Results and discussion

The chemical compositions of the samples are described in Table 3. The composition of all marble samples is dominated by calcium oxide with an average concentration of 72% which can be considered as strong evidence that calcite is the major mineral of these samples, moreover, it confirms the marble classification. On the other hand, the chemical compositions of granite samples approximately coincide with the worldwide average chemical composition of granite rocks^30^ Furthermore, granite sample compositions are dominated by silica (SiO_2_) and Aluminum oxide (Al_2_O_3_) with average ratios of 74% and 14% respectively which may indicate to the presence of the quartz and feldspar minerals in all granite samples under study. granites samples G.4 and G.6 may contain potassium feldspar due to the existence of potassium oxide (K_2_O) with concentrations of 4.52% and 4.7% respectively in their chemical compositions on contrast granite white Halayeb (G.4) contains sodium oxide (Na_2_O) instead of K_2_O so it can be concluded that the mineral potassium feldspar replaces potassium feldspar in this granite type.

**Table 3:** Chemical compositions of studied samples.

Chemical compounds	Materials					
	M.1	M.2	M.3	G.4	G.5	G.6
	Weight% (wt%)					
Na_2_O	–	–	0.2	–	4.21	–
Al_2_O_3_	–	–	0.36	12.86	14.99	14.61
SiO_2_	5.24	3.52	1.52	77.92	74.19	77.54
K_2_O	–	0.325	0.16	4.52	–	4.7
CaO	71.42	72.40	73.61	3.26	4.22	3.03
TiO_2_	–	–	-	0.36	0.24	-
FeO	–	–	-	-	1.55	-
Cr_2_O_3_	–	–	-	0.07	-	-
MnO	–	–	-	0.06	-	-
CuO	–	–	-	0.1	-	-
L.O.I	23.34	23.75	24.15	0.85	0.6	0.12

**Fig. 2:**
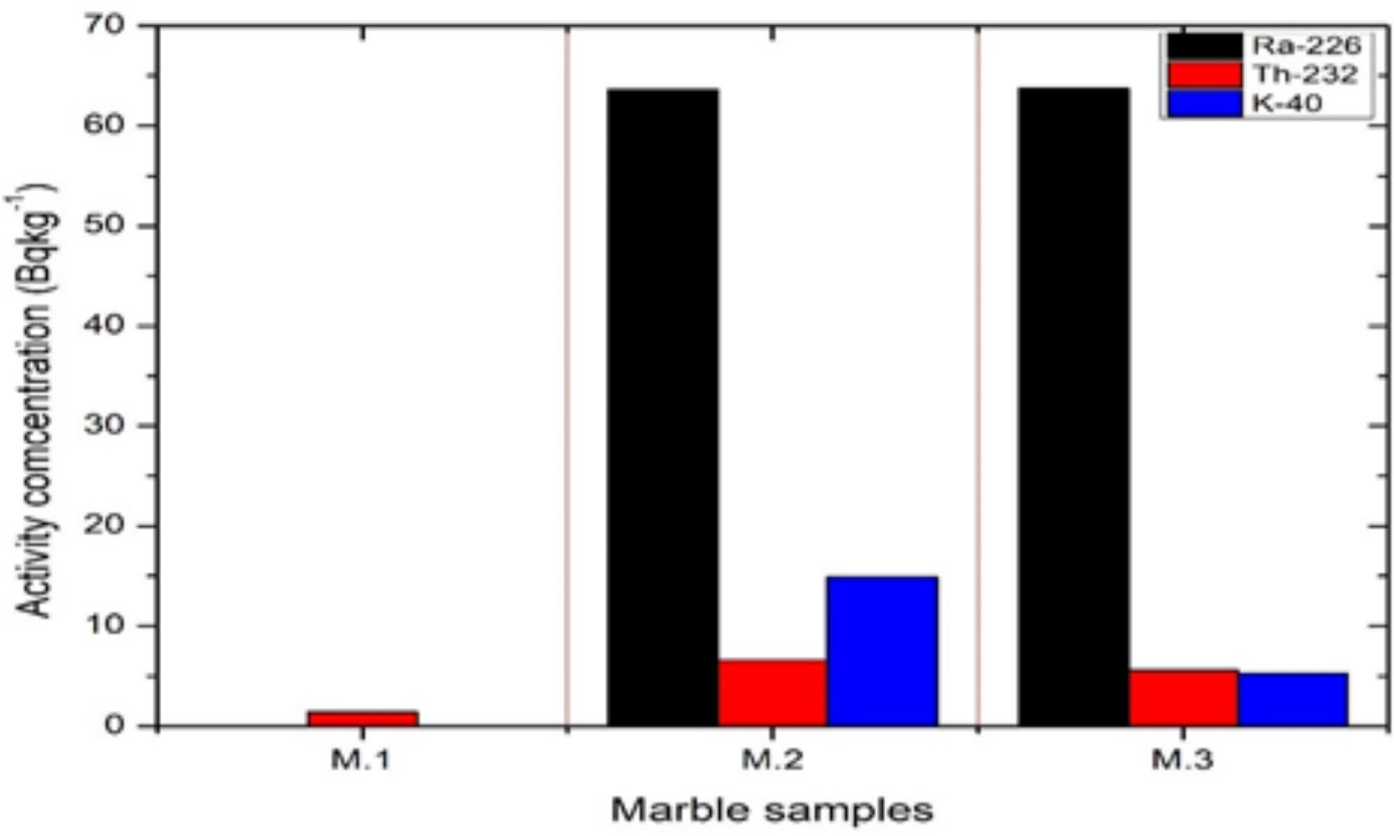
The activity concentrations of Granite samples for ^226^Ra, ^232^Th, and ^40^K.

**Fig. 3:**
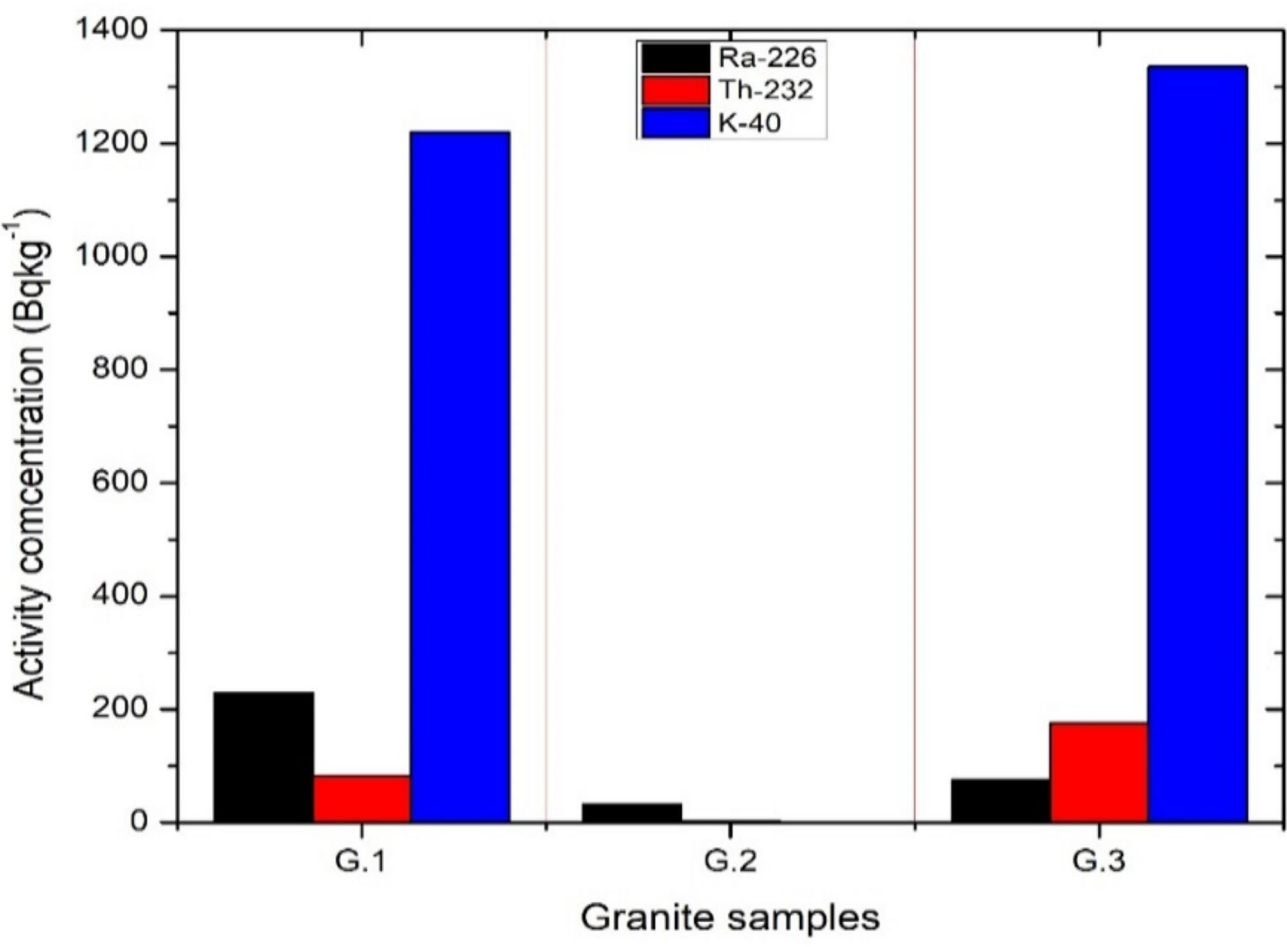
The activity concentrations of Marble samples for ^226^Ra, ^232^Th, and ^40^K.

Table 4 manifests the activity concentrations of interested nuclides of all investigated samples, all marble samples show low radioactivity with average activity concentrations of 20, 4.51, and 6.72 Bqkg^− 1^ for ^226^Ra, ^232^Th, and ^40^K respectively. Granite samples have a relatively higher activity concentration except for G.2 (white Halayeb). The measured specific activities of G.1 were 229.2,82.83 and 1220.14 Bqkg^− 1 for 226^Ra, ^232^Th, and ^40^K respectively. The activity concentrations of G.3 were 75, 175.10, and 1235 for ^226^Ra, ^232^Th, and ^40^K respectively, while G.2 recorded low radioactivity levels of ^226^Ra, ^232^Th, and ^40^K as shown in Table 4 and. Figures 2 and 3 illustrate the distribution of specific activities of all samples investigated. Table 5 provides a comparison of the average values of ^226^Ra, ^232^Th, and ^40^K specific activity in some marble and granite samples with those from other countries. The results of the present study are in good agreement with other research, Moreover, the results from comparison studies emphasize that Granite rocks on average have higher uranium and thorium content than marbles and elevated concentrations of ^40^K.

**Table 4:** Samples-specific activities (Bqkg^− 1^).

Samples		Activity concentration of interested radionuclides		
		C_Ra_	C_Th_	C_K_
Marble	M.1	U.L.D	1.5 ± 0.10	U.L.D
	M.2	64 ± 50	7 ± 0.4	15 ± 1.5
	M.3	64 ± 6.8	6 + 0.3	5 ± 0.50
Granite	G.1	229 ± 10	83 ± 7.0	1220 ± 10
	G.2	33 ± 2.5	3 ± 0.50	U.D.L
	G.3	75 ± 4.0	175 ± 10	1235 ± 20

**Table 5:** Comparison of specific activity (Bq/kg) with previously published results for marble from different countries.

Rock type	Countries	C_Ra_	C_Th_	C_K_	References
Marble	Turkey	10–92	4–122	28–676	[30]
	Nigeria	2	1	7	[31]
	Algeria	18	23	310	[32]
Granite	Italy	64	91	1206	[33]
	India	119	172	1082	[34]
	Greece	77	91	929	[35]
	Holland	162	490	1540	[36]

Table 6 summarizes the calculated radiological hazard parameters of samples under investigation. The annual effective absorbed doses of all marble samples are under the recommended upper level of 450 µSvy^− 1^, in contrast, the granite samples G.1 and G.3 record the highest annual effective absorbed doses of 1007.64 µSvy^− 1^ and 987.14 µSvy^− 1^ respectively while G.2 (White Halayeb) samples have a lower level of an annual effective dose of 80.13 µSvy^− 1^ beneath the upper recommended level of the annual effective dose.

**Table 6:** The annual effective absorbed doses and radiological hazards indices of the samples under study.

Radiation hazard parameters	The recommended upper limit	Samples					
		Marbles			Granites		
		M.1	M.2	M.3	G.1	G.2	G.3
AEDE_indoor_ (µSvy^− 1^)	450	4.42 ± 0.36	157.98 ± 13.82	152.68 ± 15.23	1007.64 ± 147.19	80.13 ± 6.83	987.14 ± 45.16
H_ex_	1	0.01	0.20	0.19	1.19	0.10	1.14
H_in_	1	0.01	0.37	0.37	1.81	0.19	1.34
Ra_eq_ (Bqkg^− 1^)	370	1.94	74.27	72.09	441.59	37.85	428.05

**Fig. 4:**
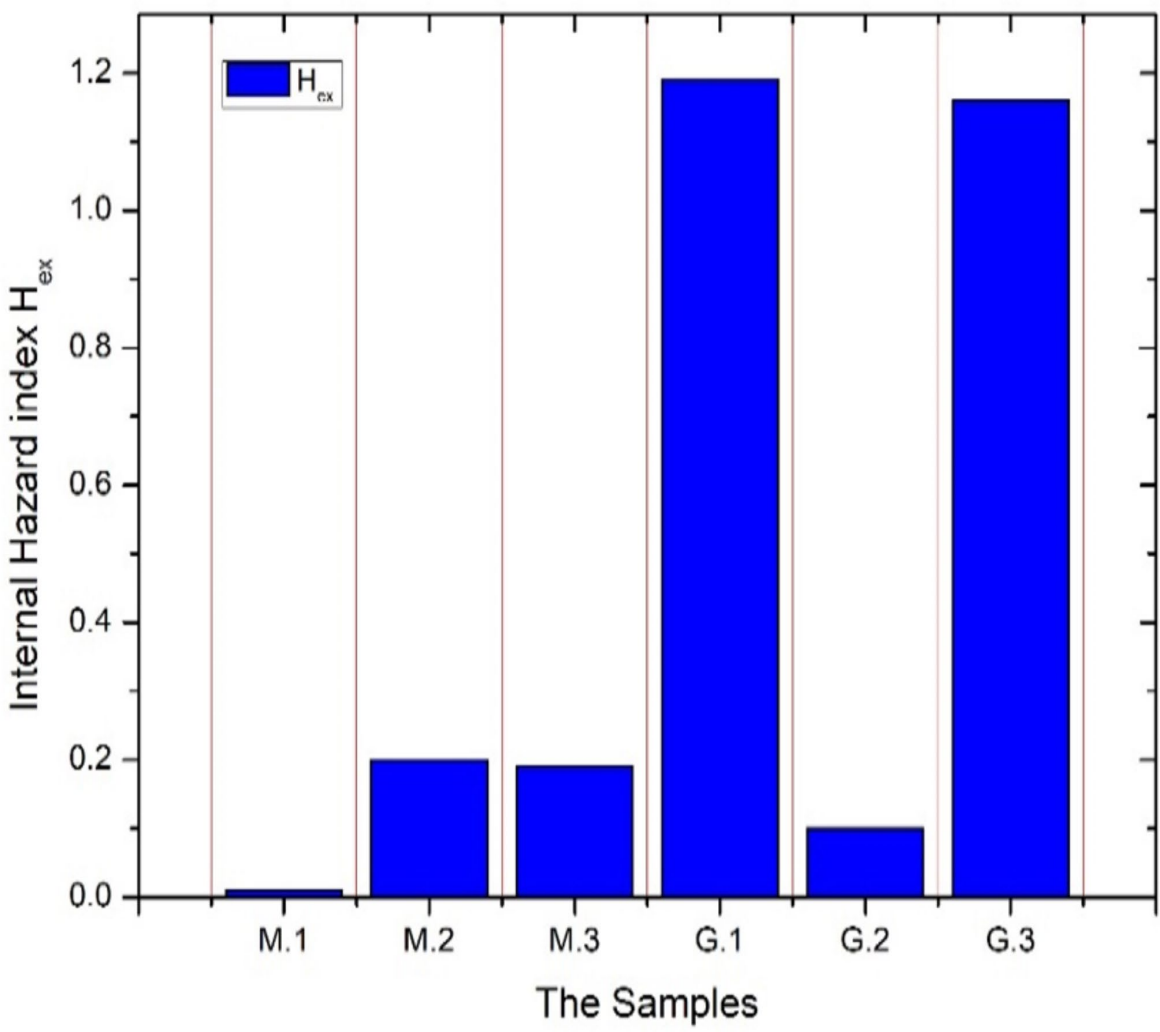
The external hazard indices of materials under study.

The external hazard indices of the investigated samples are illustrated in Fig. 4, as discussed before all marble types have an external index less than the unity which means the marbles are radiologically from the external radiation exposure point of view that can be interpreted as all marble’s samples do not have any significant content of potassium and have a neglected trace amount of ^238^U and ^232^Th, the calcite mineral dominates their geochemical composition which is radiologically safe and their possible metamorphic formation from pure limestone^37^.

**Fig. 5:**
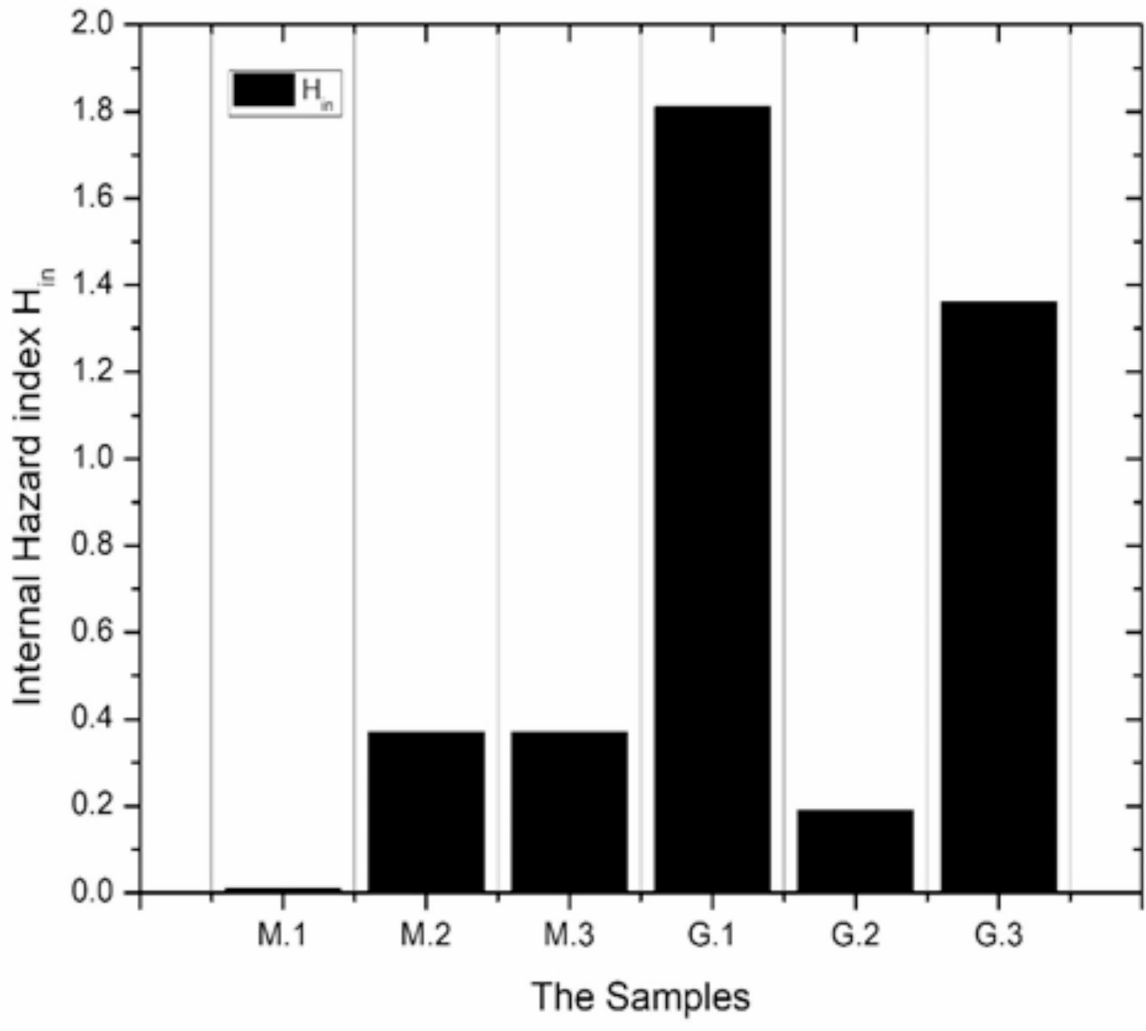
The internal hazard indices of materials under study.

White Halayeb granite (G.2) is also radiologically safe as it records an external hazard index of 0.1 while the other two types of granites G.1 (Gandola) and G.3 (Red Aswani) exhibit external hazard indices of 1.81 and 1.36 respectively so they can be considered radiologically unsafe from the external exposure point of view. The previous result can be explained as G.2 contains neglected concentrations of ^238^U and ^232^Th, moreover, it contains sodium oxide instead of potassium oxide in its composition which also indicates the presence of sodium feldspars in G.2 instead of potassium feldspars in G.1 and G.3 that causes a reduction in annual effective absorbed dose equivalent and external hazard index of white Halayeb due to the absence of ^40^K term in the external exposure hazard calculations. Furthermore, if the ^40^K activity concentrations of G.1 and G.3 are set to zero, their external hazard indices will be decreased under the recommended levels of unity to be 0.94 and 0.88 respectively so it can be concluded that potassium feldspar mineral in G.1 and G.3 granite types has significant contribution to external exposure hazards of these types of rocks (^40^K contributes to an average of 22% of the external exposure hazard), this conclusion can be reinforced by that the potassium feldspar mineral can be potentially considered as mineral bearing uranium^38^. Moreover, the probability that NORM (naturally occurring materials) may be found in granite is high because granite is an igneous rock that formed from the solidification of the earth’s molten magma hence the chance of contamination by NORM is elevated than of marble that formed by metamorphic processes. ^238^U series which is represented by ^226^Ra activity concentration in external exposure calculations has also a substantial contribution to the external hazard index of G.1 of about 51% and ^232^Th series contributes to about 27% of external exposure hazard. On the other hand, ^238^U series and ^232^Th series contribute to 18% and 59% of external exposure hazard respectively for G.3 The internal hazard indices for all types of marble are far below the unity that is expected for materials that possess insignificant ^238^U and ^232^Th activity concentrations. from Eq. 4 C_Ra_ and C_Th_ dominate the internal hazard index. The internal hazard indices for granite types G.1 and G.3 are 1.81 and 1.34 respectively while the internal hazard index of G.2 is 0.19 hence G.2 is the only radiologically safe granite type of those under investigation. Figure 5 describes the distribution of the internal hazard index of all investigated samples. Finally, the radium equivalents of all samples are calculated, and it is found that marble samples and G.2 (White Halayeb) radium equivalents are under the limit of 370 Bqkg^− 1^. The granite samples G.1 and G.3 radium equivalents are 441.59 and 420.35 Bqkg^− 1^ respectively. This result coincides with all stated before conclusions.

As per the previous discussion, it can be deduced that All marble types exhibit low radioactivity levels under the safe radiological level for all radiological hazard parameters like Annual effective dose equivalent, external hazard index, internal hazard index, and radium equivalent due to the domination of the clean calcite mineral and the metamorphic formation of these rocks from limestone under sediments or at igneous intrusion points reduce the probability of contamination with the radioactive materials of inner earth. On the other side, the two Granite types with potassium feldspar mineral ( Gandola and Red Aswani ) are radiological unsafe concerning all hazard parameters stated before because of the ^40^K content that is included in potassium oxide of potassium feldspar mineral furthermore the potassium feldspar mineral is potentially uranium bearing so the found concentration of NORM may be accumulated in this mineral, while the granite kind with sodium feldspar ( White Halayeb) is completely radiological safe due to the absence of potassium feldspar in its geological structure. Furthermore, building materials with a large content of potassium could represent radiological hazard due to the natural abundance of ^40^K radionuclide with an isotopic abundance of 0.0117%^39^ hence it is important to calculate the critical concentration of potassium that makes the building materials radiologically unsafe due to the ^40^K only. From Eq. 3 and Eq. 4 it can be concluded that the critical concentration corresponds to the ^40^K specific activity of 4810 Bqkg^1^ so by simple calculation, this concentration was found to be 13.2% for potassium element and 15.9% for potassium oxide.

## Conclusion

The present study reveals that Marbles and granites without potassium feldspar minerals in their geological structure are radiologically safe and can be used as building materials or decoration tiles safely. Potassium feldspar is a uranium-bearing mineral and includes a raised content of potassium leading to an increase in radiation exposure from these types of rocks due to ^40^K. It is recommended that an effective radiological investigation procedure be established and enforced by regulatory bodies to filter the newly quarried rocks before considering them as safe building units, especially potassium feldspar granites.

## Data Availability

The datasets used and/or analysed during the current study available from the corresponding author on reasonable request.
